# Mechanical Characterization of the Erythrocyte Membrane Using a Capacitor-Based Technique

**DOI:** 10.3390/mi15050590

**Published:** 2024-04-28

**Authors:** Doriana Dorta, Carlos Plazaola, Jafeth Carrasco, Maria F. Alves-Rosa, Lorena M. Coronado, Ricardo Correa, Maytee Zambrano, Braulio Gutiérrez-Medina, Erick Sarmiento-Gómez, Carmenza Spadafora, Guadalupe Gonzalez

**Affiliations:** 1 Facultad de Ciencias Naturales, Exactas y Tecnología, Universidad de Panamá, Panama City 06001-01103, Panama; doriana@indicasat.org.pa; 2 Centro de Biología Celular y Molecular de Enfermedades, Instituto de Investigaciones Científicas y Servicios de Alta Tecnología (INDICASAT AIP), Ciudad del Saber, Panama City 1843-01103, Panama; jcarrasco@indicasat.org.pa (J.C.); mariaalves@indicasat.org.pa (M.F.A.-R.); lcoronado@indicasat.org.pa (L.M.C.); rcorrea@indicasat.org.pa (R.C.); cspadafora@indicasat.org.pa (C.S.); 3 Facultad de Ingeniería Mecánica, Universidad Tecnológica de Panamá, Panama City 0819-07289, Panama; carlos.plazaola@utp.ac.pa; 4 Facultad de Ingeniería Eléctrica, Universidad Tecnológica de Panamá, Panama City 0819-07289, Panama; maytee.zambrano@utp.ac.pa; 5 Centro de Estudios Multidisciplinarios en Ciencias, Ingeniería y Tecnología (CEMCIT-AIP), Panama City 0819-07289, Panama; 6 Advanced Materials Division, Instituto Potosino de Investigación Científica y Tecnológica A. C. (IPICYT), San Luis Potosí 78216, Mexico; bgutierrez@ipicyt.edu.mx; 7 Departamento de Ingeniería Física, División de Ciencias e Ingenierías, Campus León, Universidad de Guanajuato, Guanajuato 37320, Mexico; esarmiento@fisica.ugto.mx

**Keywords:** capacitor, elasticity, erythrocyte, mechanical properties, stiffness, stiffness constant, optical tweezers, microfluidics

## Abstract

Pathological processes often change the mechanical properties of cells. Increased rigidity could be a marker of cellular malfunction. Erythrocytes are a type of cell that deforms to squeeze through tiny capillaries; changes in their rigidity can dramatically affect their functionality. Furthermore, differences in the homeostatic elasticity of the cell can be used as a tool for diagnosis and even for choosing the adequate treatment for some illnesses. More accurate types of equipment needed to study biomechanical phenomena at the single-cell level are very costly and thus out of reach for many laboratories around the world. This study presents a simple and low-cost technique to study the rigidity of red blood cells (RBCs) through the application of electric fields in a hand-made microfluidic chamber that uses a capacitor principle. As RBCs are deformed with the application of voltage, cells are observed under a light microscope. From mechanical force vs. deformation data, the elastic constant of the cells is determined. The results obtained with the capacitor-based method were compared with those obtained using optical tweezers, finding good agreement. In addition, *P. falciparum*-infected erythrocytes were tested with the electric field applicator. Our technique provides a simple means of testing the mechanical properties of individual cells.

## 1. Introduction

Historically, the etiology of diseases has been studied in terms of genetic disorders or biochemical dysfunctions. However, ever since the emergence of biomechanics and biophysics as fields of study in the 1960s, experimental techniques have been developed to investigate the mechanical properties of cells, membranes, and organelles. Slight variations in a cell’s physical properties can trigger pathological states [1,2]. External factors like electromagnetic exposure and mechanical stress can alter cellular functions and contribute to disease development [1,3,4,5]. This understanding of cellular mechanics is being increasingly used to explore various diseases at the cellular level. Examples include cancer [6,7,8], sickle cell anemia [2,9], arthritis and vascular disease [10,11], heart disease [12], skeletal disorders [13], or malaria [14,15,16].

The most used techniques for studying cell membrane mechanics include micropipette aspiration, optical tweezers, and atomic force microscopy (AFM) [17,18,19,20]. Other less traditional techniques include Magnetic Tweezers [21], Acoustic Force Microscopy (AFM) [22,23], Raman Spectroscopy [24], and Brillouin Light Scattering [25]. These methods can apply controlled forces on living cells in the pico- and nano-newton ranges, resulting in cell membrane displacements on the order of picometers and nanometers, respectively. These displacements indicate changes in the membrane’s mechanical properties, specifically its elasticity [3]. 

Despite their potential, applying these tools in cellular biology research presents substantial challenges. The high cost of the equipment itself can be a significant barrier, and the initial investment required for these advanced devices can be substantial. Furthermore, operating and maintaining these tools often require specialized expertise, adding to the ongoing costs and complexity [26,27].

These limitations spurred the development of alternative techniques using mechanics, optics, and electric fields (both uniform and non-uniform) to measure cell elasticity. For instance, Suresh et al. employed dielectrophoresis within a microfluidic chamber, enabling the deformation and analysis of multiple cells simultaneously [28]. Engelhardt and Sackmann developed the transient deformation technique, which involves applying electric fields to cells in solution [29]; this process mimics the movement of charged particles within a nonconducting material (dielectric) when placed between the electrodes of a capacitor, allowing the manipulation and analysis of cells. In addition, Sun and Lin further highlighted the cost-effectiveness of utilizing microfluidics for dielectrophoretic cell studies [30].

The transient deformation method relies on the electrical conductivity of the cellular medium. When an alternating electric field is applied on a cell surrounded by a liquid medium, a phenomenon called Maxwell–Wagner polarization occurs. This essentially creates temporary electric dipoles within the cells. The resulting polarization field then attracts these dipoles toward one of the electrodes. As the voltage increases, the cells experience a pulling force that stretches them, particularly within a specific range of frequencies [29,31,32]. 

Regarding cellular model systems, the elastic properties of red blood cells (RBCs) are critical for the delivery of oxygen into small capillaries [33]. Therefore, any changes in the deformability properties of these cells represent a critical threat to their normal biological functions [34]. For example, the flexibility of RBCs is altered when a *P. falciparum* parasite invades the erythrocyte, causing changes in the biochemical composition of the cell membrane and distorting its flexibility [16]. 

This report describes the development of a capacitor-based technique capable of measuring the stiffness status of cells and changes that might indicate pathological conditions. This is achieved through the application of an electric field across two electrodes containing a conductive medium in which the biological sample is placed. Here, the mathematical formulations, the development of the electrical force applicator, and the experimental setup used to evaluate cell deformation are described. 

The validation of the method is done through the analysis of the stiffness of RBCs with the capacitor technique and contrasting those with the results obtained by measuring cellular stiffness using an optical trap. The results presented here show the applicability of an affordable system to evaluate the mechanical properties of individual cells. 

## 2. Force Applied on Individual Cells Using the Capacitor-Based Method 

**Figure 1:**
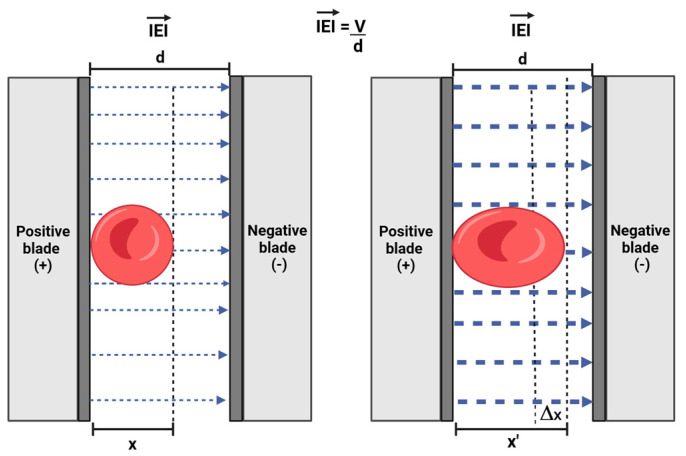
**Diagram of an erythrocyte under an electric field**. Low voltage creates Maxwell–Wagner polarization (thin arrows), and high voltage (thick arrows) creates elongation. $\left|\vec{E}\right|$ is the magnitude of the electric field; d = distance between electrodes; x = non-elongated RBC diameter (µm); x’ = elongated RBC diameter (µm); Δx = elongation. Image created with BioRender.

The capacitor-based technique relies on the principle that applying an electric voltage across the electrodes induces a deformation in the shape of the red blood cell (Figure 1). This behavior can be understood by the so-called Maxwell–Wagner polarization (or interfacial polarization) effect. Under an external high-frequency (~MHz) electric field, E, the frequency-dependent dielectric responses of the two media involved (cytoplasm and external medium) lead to the accumulation of electrical charge at the poles of the cell. Therefore, the cell behaves as an electric dipole, experiencing a mechanical force under the externally applied field [29]. This effect is related to dielectrophoresis, where polarizable particles translocate under an externally applied nonuniform electric field. 

The voltage applied to the electrodes generates an electric field between them. The strength (magnitude) of this electric field can be approximated by the following equation:

(1) $$\left|\vec{E}\right|=\frac{V}{d}$$

where $\left|\vec{E}\right|$ is the magnitude of the electric field, V represents the voltage, and *d* is the distance between the plates or electrodes. 

By integrating the Maxwell stress tensor over the surface area of the cell, A, and by performing the time-averaging of the electric field, Engelhardt and co-workers [29] proposed a formula to determine the magnitude of the mechanical force, F, that causes a cell to elongate:

(2) $$F=0.25\times \varepsilon_{0}\varepsilon E^{2}A$$

where $\varepsilon_{0}$ and ε(=78) [35] are the permittivity of the vacuum and the relative permittivity of the biological sample, respectively. In our experiments, we estimate *A* = 10 ${\mu m}^{2}$. Upon the application of the electric field, the erythrocyte experiences a force that elongates its shape along the direction of the field. Internal forces are generated within the erythrocyte membrane that counteract the external forces exerted by the electric field until the cell reaches an equilibrium state. Elongation can be quantified by subtracting the cell diameter after deformation from that of the initial condition. Finally, the slope of the force vs. elongation relationship (in the linear regime) provides a measure of the cell stiffness.

The design and construction of the electric force applicator, for mechanical characterization, based on the capacitive method, which involves the application of an electric field to two parallel plates, is described below, as well as the preparation of the biological samples.

## 3. Conceptualization of the Electric Force Applicator

A simple design for a microfluidic capacitor, also referred to as the electric force applicator, consists of two parallel electrodes separated by a microchannel, where the targeted gap is in the micrometer range. For this purpose, we used two razor blades (made of stainless steel) firmly secured onto a microscope glass slide with double-sided Scotch tape, facing each other and creating a microchannel with an intended separation of ~45 µm.

**Figure 2:**
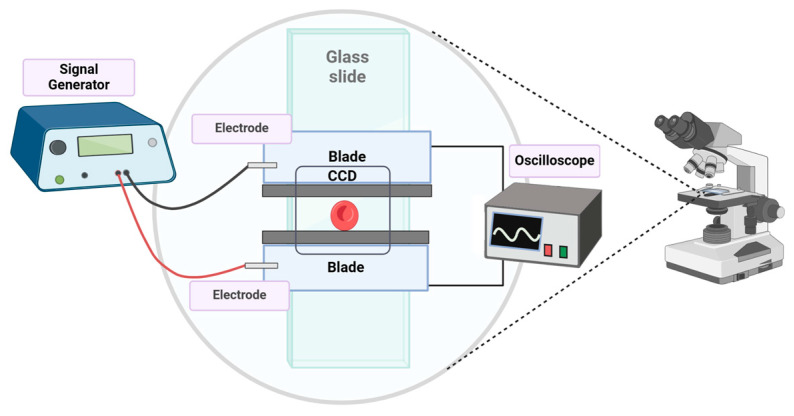
**Setup of the electric field applicator.** The microscope slide with the attached electrodes is placed on top of the microscope stage, allowing the microscope camera to take pictures of the cellular deformation caused by the electric field. The image was created with Biorender.

**Figure 3:**
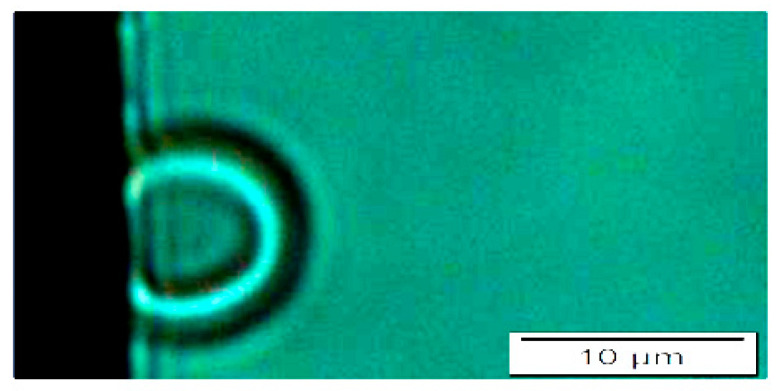
**Effect of voltage applied to the microchannel on erythrocyte polarization: low voltage**. Representative image of an erythrocyte polarized in response to a low voltage applied to the electrodes in the microchamber. The black border is one of the electrodes. Magnification: 100×.

This assembly was fabricated on a microscope slide, as illustrated in Figure 2. An external signal generator or power source (Agilent 33220A 20 MHz, Santa Clara, CA, USA) connected directly to the capacitor plates enabled the application of an electric field in the gap region between the electrodes. The equipment included an electric signal meter (Agilent Technologies DSCO-X 2014 oscilloscope, Santa Clara, CA, USA) connected in parallel with the signal generator to measure the actual voltage applied, considering the potential signal attenuation along the transmission lines and the dielectric material. It is important to note that the strength (magnitude) of the applied electric field is directly dependent on the dimensions of the microchannel. The experiments were designed so that the biological samples were placed on the electric force applicator and observed with a microscope (Olympus BX53, Tokyo, Japan), a DP37 camera, and a computer (Figure 3). Microscope images were processed using CellSens 1.17 (OLYMPUS, Tokyo, Japan).

## 4. Materials and Methods

### 4.1. Biological Dielectrics

In these experiments, the biological samples were suspended in one of two solutions, each with a different conductivity. The one that helped the cells respond better to the electric fields, allowing them to elongate, was chosen for the electrical field exposure.

### 4.2. Experimental Media

#### 4.2.1. Citric Acid, Sodium Citrate, and Dextrose (ACD) Solution

This solution was prepared by dissolving 0.15 M citric acid (C_6_H_8_O_7_), 0.087 M sodium citrate (Na_3_C_6_H_5_O_7_), and 0.122 M dextrose in sterile water to reach the desired volume. It was adjusted to a range between 7.2 and 7.4 to maintain physiological pH. Following pH adjustment, the solution’s conductivity was measured to ensure a value of 15 mS/cm. Finally, the ACD solution was sterilized by filtration through a 0.2 μm filter (Acrodisc^®,^ PALL, Port Washington, New York, NY, USA) and stored at 4 °C until use [31].

#### 4.2.2. Measuring Solution

This solution contained 250 mM mannitol, 30 mM glucose, and 1 mM calcium chloride (all from Sigma-Aldrich, St. Louis, MO, USA) dissolved in water. Unlike the ACD solution, the pH of the measuring solution (approximately 5.39) was left unadjusted to preserve its original conductivity of 301 µS/cm [31].

#### 4.2.3. Erythrocytes

Human blood was collected from a pool of volunteers who signed informed consent forms. The protocol was approved by the bioethical committee of The Gorgas Memorial Institute of Health Sciences in Panama with Number 0001–2017. A total of 9 mL of blood was collected in a tube containing 1 mL of anticoagulant CPDA (Citrate Phosphate Dextrose Adenine) (Sigma-Aldrich, St. Louis, MO, USA). The blood sample was centrifuged at 2500 rpm for 8 min at 10 °C. This centrifugation process separated the blood components into layers. The intermediate layer, rich in white blood cells, was discarded. The remaining pellet, enriched with red RBCs, was resuspended in RPMI medium (Sigma-Aldrich, St. Louis, MO, USA) and centrifuged again. This washing step was repeated 2–3 times to remove residual plasma and white blood cells. After the final centrifugation, the supernatant was discarded, and the pellet containing the purified RBCs was resuspended in an equal volume of fresh RPMI medium. RBCs were then stored at 4 °C until use. This suspension was named RBC50/50. 

#### 4.2.4. Sample Preparation

The sample consisted of 100 µL of RBC 50/50 diluted in 300 µL of ACD (RBC-ACD). We mixed 30 µL of RBC-ACD in 400 µL of measuring solution to obtain the biological sample. We used 15 µL of RBC-ACD in each determination. It is worth noting that samples retained the integrity of the cell plasma membrane for only 15 min due to the measuring solution being mildly acidic (pH 5.39). This limitation imposed a minimal time frame for conducting the experiment.

For optical tweezers experiments, RBCs were incubated for 1 h with a well-mixed suspension of 3 µm polystyrene beads, enabling beads to adhere to the cell membrane. After incubation and deposition in the microscope slide, some cells attach one end to the glass surface, and the opposite end has a single bead attached to cell surface, creating a stable “cell–bead–glass” configuration. These cells are the target for deformation [36].

#### 4.2.5. Infected Erythrocytes

*P. falciparum* parasites were cultivated following the method described by Trager and Jensen [37] with some modifications. Human O+ RBCs were used in a culture media composed of RPMI 1640 supplemented with 10% human serum. The infected culture was incubated at 37 °C in a gas mixture containing 90% nitrogen, 5% carbon dioxide, and 5% oxygen. RBCs were sampled at a 2% hematocrit (volume percentage of RBCs). To ensure the homogeneity of the parasite’s stage, they were synchronized with sorbitol, which inhibits the growth of late stages of *P. falciparum* by affecting their permeability, leaving behind only early-stage parasites. 

#### 4.2.6. Isolation and Preparation of *P. falciparum*-Infected Erythrocytes (iRBC)

Using the paramagnetic properties of the so-called “malaria pigment” (hemozoin) [38], a magnetic separation method was employed to isolate the iRBCs containing mature trophozoites and schizonts, following the protocol outlined by Coronado et al. [39].

Once the iRBCs were isolated in the trophozoite or schizont stage, 20 μL of RBC 50/50 was taken and placed in 10 μL of ACD (iRBC-ACD). To protect the fragile iRBCs during electric field application and to prevent non-specific adhesion to the microchannel, 10 μL of 1 mg/mL bovine serum albumin (Sigma-Aldrich) was added, and the mixture was incubated for 5 min. Then, 30 μL of the iRBC-ACD solution was placed in 30 μL of the measuring solution. Finally, 15 μL of this mixture was placed in the microchannel previously treated with 10 μL of measuring solution.

#### 4.2.7. Capacitor-Based Method: Experimental Considerations and Frequency

The experimental setup consisted of the microfluidic chamber with the biological sample inside, placed between electrodes. A sinusoidal voltage with varying amplitudes (0 to 18 Vpp) was applied at a frequency of 1 MHz. It should be noted that at frequencies exceeding 10 MHz, both the suspending solution and the RBCs exhibit primarily an insulating behavior. Additionally, RBCs can move freely within the medium at these high frequencies, hindering the observation of the desired cell elongation. When frequencies drop below 0.1 MHz, the mechanical response of RBCs involves rotation along an axis parallel to the electrodes. This rotational motion also prevents the observation of elongation. The frequency range of 0.1 MHz to 1 MHz offered several advantages. Within this range, the conductivity of the viscous medium (including the ACD and measuring solutions) differs significantly from that of the RBCs. Furthermore, as illustrated in Figure 3, the Maxwell–Wagner polarization becomes significant in this range [29], allowing for the separation of charges in the RBCs, causing them to attach on one of their sides to the oppositely charged pole when an electric field is applied.

**Figure 4:**
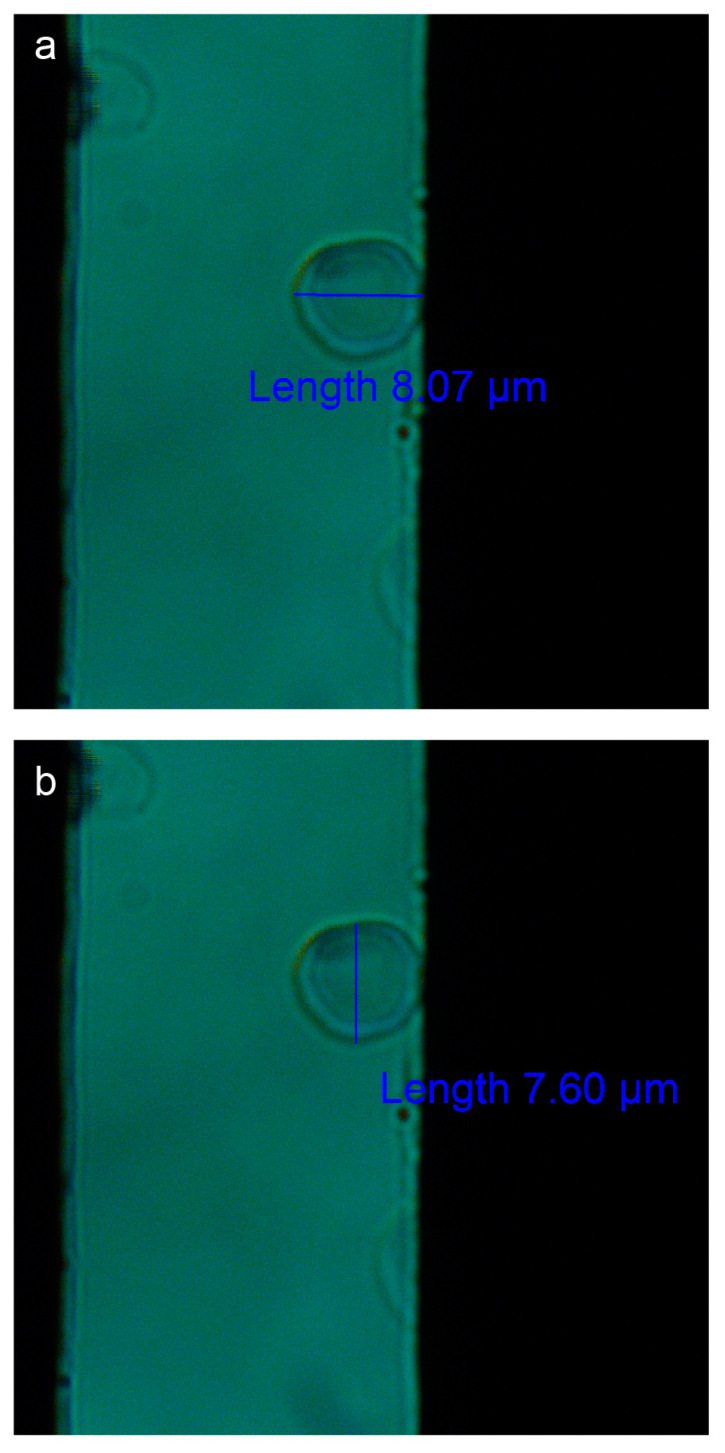
**Measurement of the erythrocyte**. The image depicts how the erythrocyte was measured along the horizontal *x*-axis (**a**) and vertical *y*-axis (**b**). The black borders represent the electrodes. Images were taken with a light microscope using a 100× objective.

To measure the changes in cell shape induced by an increasing voltage, the dimensions of the erythrocytes were measured along their horizontal and vertical axes (Figure 4a and Figure 4b, respectively).

Once the system was assembled, a sinusoidal voltage with varying amplitudes from 0 to 18 Vpp was applied. The experiment was carried out at 1 MHz, at room temperature (~22 °C). 

### 4.3. Measuring Force Using an Optical Trap

To validate our capacitor-based technique, we used optical tweezers (or optical traps) to evaluate RBC. Optical traps provide excellent means of exerting and measuring forces in the pN range, although they are complex and difficult to implement. 

The optical manipulation phenomena rely on the interaction of a dielectric particle with a highly focused beam, effectively producing a trapping external potential for the particle. For a spherical dielectric particle in a Gaussian beam, the trapping potential can be modeled as a parabolic potential, and thus the restoring force applied to the particle corresponds to Hooke’s law:

F = −kΔx(3)

where k is the trap stiffness, and Δx is the displacement from the center of the trap. A meticulous calibration process, usually based on analyzing the Brownian motion of the trapped particle, establishes the trap stiffness of the optical trap. This essentially quantifies the strength of the restoring force exerted by the trap on the displaced particle and depends on the particle’s size and index of refraction and on the laser power used. Once the trap stiffness is determined, this probe can be used to determine forces using Equation (3).

We have previously described how we implement the optical tweezers methodology in our group [40]. Briefly, our optical tweezers equipment consists of a commercial, modular optical trapping system (MMI GmbH, Dresden, Germany) coupled to an inverted Olympus IX83 microscope. Trapping light comes from a solid-state laser of wavelength λ = 1070 nm and 8 W maximum power. Typical laser power used for trapping: 0.8 W. Laser focusing is produced by an oil-immersion, 100× objective with numerical aperture NA = 1.2. Calibration of the trap is performed using the oscillatory drag method [36]. Typical stiffness of the optical trap: k = 64 pN/μm. To minimize optical trapping stiffness variation due to changes in trapping height, we used beads of size 3 µm, comparable to the width of RBCs, and selected cell-bead geometries where cells were found with their long axis nearly parallel to the glass surface and the bead attached to the rim of the cell. This geometry ensured sample-to-sample reproducible conditions, with trapping height ~ 3 µm (distance from bed center to glass surface).

To exert mechanical force on RBCs, a 3 μm polystyrene bead was attached to the cell using an incubation protocol designed to reduce the electrostatic repulsion between the particle and the cell [36]. Then, the particle was trapped in the optical tweezers to produce a deformation on the cell and quantify the restoring force. 

Cells targeted for deformation corresponded to cells in which the bead was attached to one side of the cell and free to move, and the opposite part of the cell was attached to the glass plate, thus allowing a one-side deformation using optical tweezers [36]. Once the particle was trapped, the position of the trap was modified to produce a deformation on the cell. The offset between the position of the particle and the center of the trap was used to calculate the restoring force using Equation (3). This procedure was repeated several times to recover an elongation vs. restoring force curve. To maintain mechanical equilibrium (Newton’s Third Law), the glass plate was expected to exert a force on the cell equal to the force applied by the optical tweezers. However, only the force from the tweezers was quantified.

## 5. Results

### 5.1. Cell Deformation under Electric Fields

To characterize the deformation behavior of RBCs, experiments were performed using an electric force applicator, maintaining a constant frequency of 1 MHz while gradually increasing the voltage. When the applicator is placed under a light microscope with a camera, the progressive changes in cell shape induced by the electric field at a fixed frequency can be recorded. 

The voltage applied to each sample varied from 0 to ~4.5 V, and cellular deformations were tracked at a specific point on the cell’s circumference relative to the *x*-axis, as seen in Figure 4a. Table 1 shows results from a representative experiment, displaying the applied voltages and the electric field intensities to a single cell. The radii of each cell in two perpendicular directions are also indicated (Figure 4). We observed that as the voltage increased, so did the *x*-axis radii, and these changes were inversely proportional to those observed along the RBC’s *y*-axis radii. 

**Figure 5:**
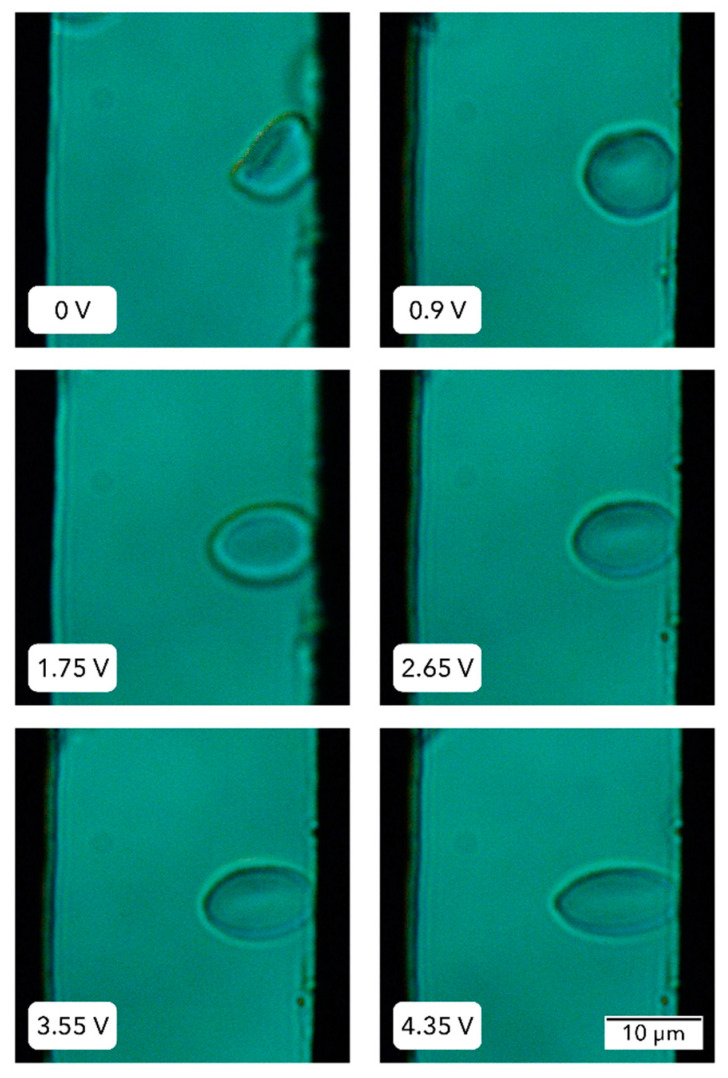
**RBC deformation behavior under gradually increasing voltage.** Erythrocytes were placed in the capacitor chamber, and the voltage applied was increased from 0 until the RBC was still elongating. This occurred at 4.35 V. Images were taken with a light microscope, using a 100× objective.

It was found that the erythrocyte could elongate up to 1.4 times when the maximum voltage was applied. Any voltage increase beyond this value did not significantly change the dimensions of the RBC. The voltage at which maximum elongation is observed is here referred to as “maximum voltage”. We performed analyses in the linear force vs. extension relationship. Figure 5 illustrates another representative experiment. In that case, the particularities of the erythrocyte could withstand a greater deformation with increased voltages. The experiment was repeated on nine independent erythrocytes.

### 5.2. Red Blood Cell Stiffness 

We calculated the force exerted on each cell when the electric field was applied, using Formulas (1) and (2). The stretching force exerted on the erythrocyte was found to be in the range of piconewtons (pN). Thus, we evaluated the association between force (pN) exerted on each cell and cell elongation (µm). We define cell elongation as the distance increase in cell width upon the application of the external electric field. The slope of the linear regression of the paired values allowed us to determine the cellular stiffness (pN/µm). High r^2^ values (r^2^ > 0.9) suggest that the determined magnitude accurately represents the rigidity of each RBC. 

**Figure 6:**
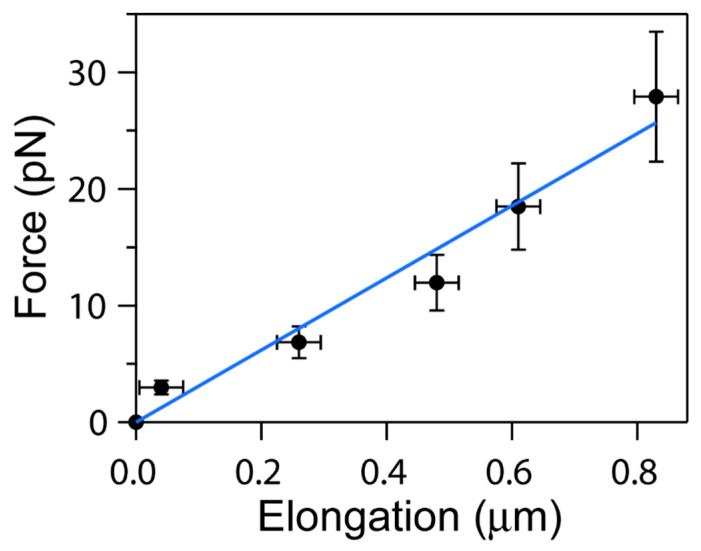
**Relationship between cellular displacement and applied force in RBCs subjected to electric fields**. Applied forces (pN) induced cellular elongations (µm) along the *x*-axis of the RBC. A linear fit to data yields a slope: 30.9 ± 1.6 pN/μm, equal to the elastic constant of the cell. This is a representative graph of three independent measurements of RBCs derived from the same donor. Error bars: see the main text.

In addition, when the stiffness values from three independent RBC measurements from the same donor were compared to each other, we found no significant differences between them (ANOVA *p* > 0.05), indicating that this methodology yields reproducible results. Figure 6 shows a representative experiment using an RBC subjected to electric field forces, together with a linear regression to experimental data. Error bars in the measurements of force are evaluated from Formula (2), using propagation of error, where the main uncertainty is associated with the distance between electrodes (δd~5 μm). For cellular elongation, the uncertainty is provided by half the pixel size in the microscope images (~35 nm).

The above analysis was conducted for nine independent erythrocyte samples, collected from different donors. The stiffness values for these cells were averaged, from where we obtained the characteristic value of erythrocyte stiffness using the capacitance method: 13.2 ± 6.8 pN/µm (average ± SD, *N* = 9). 

### 5.3. Comparative Analysis from RBC Stiffness Values Obtained from Optical Traps vs. Electric Field Devices

Optical traps are novel and high-end devices capable of manipulating dielectric particles ranging in size from nanometers to microns using highly focused laser beams to apply extremely small forces. Thus, we evaluated stiffness values from RBCs manipulated by optical traps and compared them with those obtained from the electric field device. 

**Figure 7:**
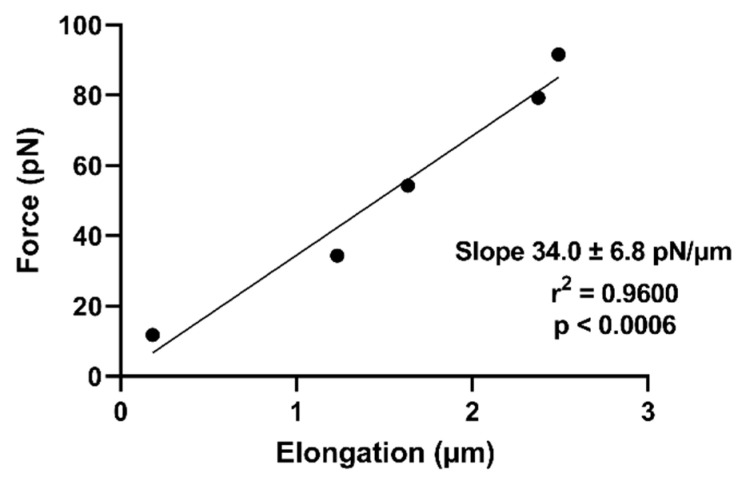
**A typical example of the relationship between cellular displacement and applied force in erythrocytes subjected to an optical trap**. Cellular elongation (µm) along the *x*-axis induced by optical tweezers was measured and plotted against the corresponding applied force (pN). The slope value obtained from linear regression analysis is indicative of cell stiffness.

**Figure 8:**
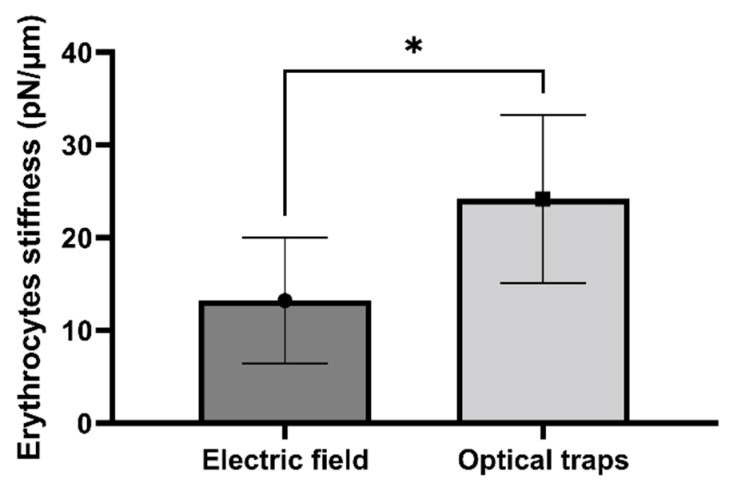
**Comparison of erythrocyte stiffness values obtained using the electric-field-capacitor-based device or optical traps**. Bars correspond to mean values for RBC stiffness obtained from electric field (dark gray) analysis and optical traps (light gray). Error bars: SD; *N* = 9. * *p* = 0.05, *t*-test (see main text).

The data in Figure 7 show applied force vs. elongation obtained from RBCs elongated by an optical trap, resulting in a cell stiffness value of 24.2 ± 9.1 pN/µm (average ± SD, *N* = 9). This result can be compared with that obtained from using electric field forces (see Figure 8). A comparison between the two methods (Student’s paired *t*-Test, with a two-tailed distribution) results in *p* = 0.05, which indicates a significant difference between the two populations. This result could be explained by the approximations used in calculating mechanical force in the two methods or by differences in the behavior of the two groups of erythrocytes tested. In any case, the agreement between RBC stiffness values is good and certainly agrees with previously published results using a variety of techniques (17, 28, 41). 

### 5.4. Falciparum-Infected Erythrocyte Stiffness

A challenging test was conducted on the microfluidic device. A very rigid cell by nature was placed in the capacitor device. Table 2 summarizes the changes in the radii of *P. falciparum*-infected erythrocytes in the late stages of intraerythrocytic infection. The data reveal no variations in any of the horizontal (*x*-axis) or vertical (*y*-axis) dimensions with applied voltages ranging from 0 to 2.8 V. It is of note that, while the elongation of a healthy RBC increased by 20% at 2.54 V, no significant deformation was achieved in the iRBC, despite the substantial increase in the voltage beyond 2.8 V. Thus, it was not possible to determine iRBC stiffness values using the capacitor-based method. Despite this apparent negative result, this finding coincides with those from Du et al., 2014 [28], who also tried to deform *P. falciparum*-infected erythrocytes without success, using microfluidics in a more sophisticated device.

**Figure 9:**
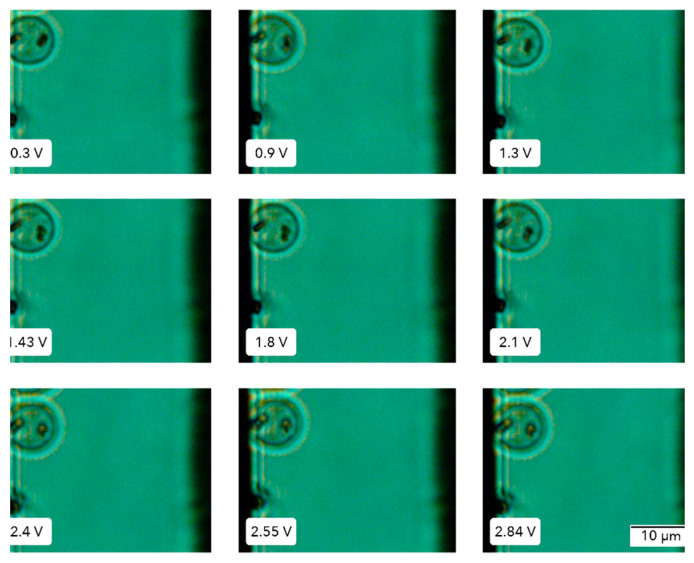
**Erythrocytes infected with *P. falciparum***. iRBC was placed in the capacitor chamber, and the voltage applied was increased from 0 to 2.85 V, at 1 MHz frequency. Images were taken with a light microscope using a 100× objective.

Figure 9 presents a sequence of images depicting iRBCs subjected to an electrical field. Unlike healthy RBCs, these cells did not exhibit significant morphological changes, such as the expected elongation response, even when exposed to increasing voltages. Interestingly, uninfected erythrocytes generally tolerated voltages above 5 V. In contrast, the levels reaching the channel dropped significantly when applying the same voltage to *P. falciparum*-infected cells, perhaps due to some intrinsic electrophysical property of the parasite.

## 6. Conclusions

This work presents a novel and cost-effective experimental method for biomechanical studies, leveraging established electrical and mechanical principles. We designed a homemade yet reliable electric force applicator to evaluate cellular stiffness. Healthy red blood cells (RBCs), known for their elasticity and minimal stiffness, were chosen to test the functionality of the device.

By applying an electric field through the capacitor-based device we were able to infer an important biomechanical property: cellular rigidity. This rigidity is reflected in the observed elongation of the RBCs under the electric field. The capacitor-based device presented here gave the stiffness value of a human erythrocyte placing it at 13.2 ± 6.8 pN/µm under the experimental conditions. This value falls within the reported range for human erythrocyte stiffness (10–50 pN/µm) according to the previous literature [17,28,40]. These findings demonstrate that our cost-effective system offers a reliable alternative for characterizing cell stiffness, yielding results comparable in magnitude to those obtained with the significantly more expensive optical tweezers.

We were unable to determine the stiffness constant of the infected red blood cells (RBCs) because of a lack of quantifiable deformation. While we explored various voltage ranges with the electric applicator, no significant changes in shape or size were observed. These findings, nonetheless, align with previous reports [28,41], confirming that *P. falciparum* infection gravely alters the rigidity of the RBC membrane, potentially contributing to the observed lack of measurable deformation under electric fields.

Interestingly, while uRBC-ACD reached voltages up to 9 V, the iRBC-ACD did not allow the voltage to reach any higher than 3.5 V, implying that the parasite modifies the electrical properties of the cell significantly, such that it causes a voltage drop in the infected sample.

Finally, regarding the capacitor-based method, it is interesting to note that the application of mechanical forces to cells by using external electric fields has been used before through dielectrophoresis as a novel technique to calibrate optical tweezers [42].

In conclusion, our technique is affordable and robust, and it does not require specialized training in scientific instrumentation. These characteristics make our work relevant, markedly in low-resource settings. The simplicity of this system suggests its potential applicability to a wider range of cell types beyond RBCs, enabling the analysis of biomechanical properties in healthy and diseased cell states. Future work should include incorporating the capacitor-based method into a microfluidic platform to achieve high throughput. We expect that the method could be applied to probe the mechanical properties of other globular cells besides erythrocytes, such as fungal spores, bacteria, and animal egg cells, among others.

## Figures and Tables

**Table 1:** Effect of the application of electric fields on the size of an RBC.

Voltage ± 0.001 (V)	Field Intensity (kV/m)	Radius-X Axis ± 0.035 (µm)	Radius-Y Axis ± 0.035 (µm)
0	0	3.87	4.06
0.865	16.6 ± 1.7	3.92	3.92
1.71	32.9 ± 3.3	4.43	3.83
2.545	48.9 ± 4.9	4.6	3.35
3.355	64.5 ± 6.4	4.92	3.17
4.16	80 ± 8.0	5.33	2.94

**Table 2:** Effect of the application of electric fields on the size of an iRBC.

Voltage ± 0.001 (V)	Field Intensity (kV/m)	Radius-X Axis ± 0.035 (µm)	Radius-Y Axis ± 0.035 (µm)
0.3	9.2 ± 0.9	2.51	3.54
0.9	20.0 ± 2.0	2.62	3.45
1.3	27.8 ± 2.8	2.4	3.52
1.4	39.2 ± 3.9	2.65	3.55
1.8	44.2 ± 4.4	2.5	3.54
2.1	55.7 ± 5.6	2.59	3.58
2.4	66.2 ± 6.6	2.7	3.52
2.5	73.9 ± 7.4	2.7	3.52
2.8	78.5 ± 7.8	2.54	3.48

## Data Availability

The original contributions presented in the study are included in the article, further inquiries can be directed to the corresponding authors.
